# Solitary verrucous plaque on the chin: hypertrophic discoid lupus erythematosus^⋆^^⋆^

**DOI:** 10.1016/j.abd.2024.06.005

**Published:** 2025-01-15

**Authors:** Carolina Viza Amorim, Maria de Lourdes Bialon Santana, Maria Leticia Cintra, Fernanda Teixeira

**Affiliations:** Department of Pathology, Faculty of Medical Sciences, Universidade Estadual de Campinas, Campinas, SP, Brazil

Dear Editor,

Hypertrophic Discoid Lupus Erythematosus (HDLE) is a unique subgroup of Cutaneous Lupus (CL) characterized by chronic, verrucous lesions that commonly affect the head and arms. It represents about 2% of all cases of chronic CL.^1^ Typical discoid lesions usually accompany HDLE. Due to its unusual presentation, HDLE requires a differential diagnosis with inflammatory, reactive, infectious, and neoplastic diseases that manifest with epidermal thickening,^2^ as well as hypertrophic lichen planus^3^ and lichen simplex chronicus.^4^ Systemic involvement is rare.^5^

HDLE has a risk of malignant transformation into Squamous Cell Carcinoma (SCC) and, although it can be difficult to diagnose, some clinical and histopathologic features may point to malignancy.^5^, ^6^

A 42-year-old woman came to consultation with a solitary U-shaped verrucous plaque, on her chin, that grew progressively over two years and now measured 48 mm in diameter (Fig. 1A). The lesion was painless, verrucous, had infiltrated, erythematous border, and hyperchromic and a little violaceous center. She denied having received any type of treatment for it. Blood tests were all normal, including Quantiferon TB, and the chest X-ray showed no changes.

**Fig. 1:**
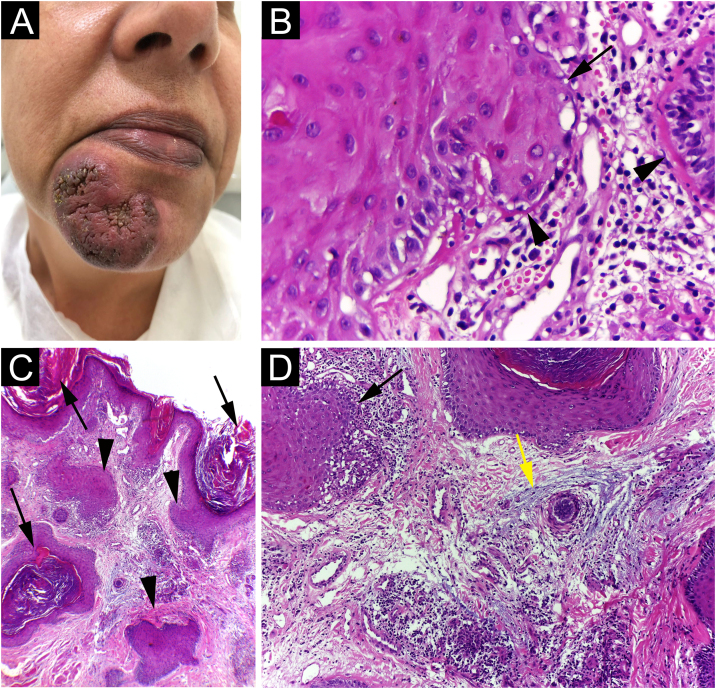
(A) Solitary verrucous infiltrated plaque with erythematous border and hyperchromic and a little violaceous color in the center; (B) The epidermis shows basal cell damage (arrows), thickening of the basal membrane (arrowheads) (Hematoxylin & eosin, ×40); (C) The epidermis shows hyperkeratosis (arrows). The dermis shows infundibular epithelium hyperplasia (arrowheads) and dermal inflammatory infiltrate (Hematoxylin & eosin, ×400); (D) The epidermis shows basal cell damage (arrows) and dermis shows mucin deposition (yellow arrows) (Hematoxylin & eosin, ×400).

During the consultation, the patient frequently manipulated the lesion. For this reason, the hypothesis of dermatitis artefacta was proposed, with a differential diagnosis of infectious granulomatous entities.

Punch biopsy showed basal cell damage, thickening of the basement membrane (Fig. 1B), hyperkeratosis, hyperplasia of the infundibular epithelium, dermal inflammatory infiltrate (Fig. 1C), and mucin deposition in the dermis (Fig. 1D). Direct immunofluorescence (Fig. 2) showed coarse granular deposits of junctional IgG (Fig. 2A) and adsorption of IgM in colloid bodies (Fig. 2B). The final diagnosis was HDLE.

**Fig. 2:**
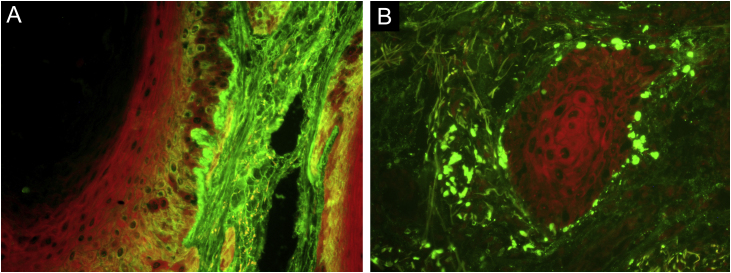
(A) Direct immunofluorescence (×400) showed junctional IgG coarse granular deposits; (B) IgM adsorption in colloid bodies.

She was treated with prednisone 40 mg/day, which was progressively tapered, and, after an ophthalmological examination, hydroxychloroquine 400 mg/day was added. Besides, intralesional triamcinolone was infiltrated monthly. Despite the treatment, after 6 months, the lesion had progressed to involve the labiomental sulcus (Fig. 3A). Hydroxychloroquine was discontinued and methotrexate 15 mg per week, folic acid and vitamin D were added to prednisone and triamcinolone. At the last appointment, 18 months after her initial visit, there was only minimal regression of the lesion (Fig. 3B).

**Fig. 3:**
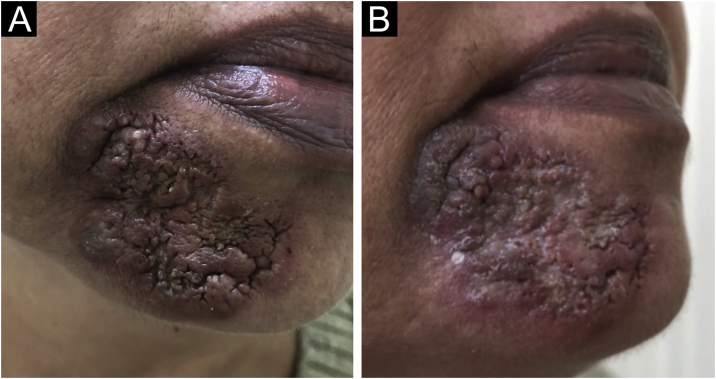
(A) Despite the treatment, after 6 months, the lesion had progressed to involve the labiomental sulcus; (B) After 21 months, minimal lesion remission could be achieved.

HDLE is an uncommon form of CL; when typical lesions of Discoid Lupus Erythematosus (DLE) coexist, the diagnosis is more easily achieved. However, solitary lesions of HDLE can mimic hypertrophic lichen planus, ostraceous psoriasis, prurigo nodularis, verruca vulgaris, lichen simplex chronicus, keratoacanthoma, SCC and even chronic cutaneous graft-versus-host disease.^5^, ^7^ Although topical imiquimod has been used as a treatment for superficial skin carcinomas, and genital warts and even as an alternative medication for CL, the induction of a lesion, clinically and histologically similar to HDLE, was described with the use of this immune response modifier.^8^

HDLE presents typical histological features of DLE, such as hydropic degeneration of the basal layer, thickening of the basement membrane, and dermal infiltration of lymphoid cells. As well as a considerable degree of hyperkeratosis, acanthosis, and papillomatosis, which may lead to the histopathological differential diagnosis of hypertrophic lichen planus, SCC, keratoacanthoma, cutaneous mycobacteriosis, and deep fungal infections.^2^, ^3^, ^4^ Furthermore, transepidermal shedding of elastotic material may be a histological feature of HDLE.^1^ Daldon et al. described three patients who developed hyperkeratotic papules with a central keratinous plug on their arms, which clinically and histopathologically resembled keratoacanthomas. The features that allowed the diagnosis of HDLE were vacuolar and lichenoid interface dermatitis, thickening of the basement membrane, and perivascular and periadnexal mononuclear infiltrate.^1^ Immunofluorescence shows deposition of IgG and IgM in a granular pattern at the dermal-epidermal junction.^5^

HDLE carries a risk of malignant transformation into SCC.^6^ It is known that **c**hronic discoid lesions also present this risk, but SCC occurs 10 times as often in HDLE.^6^ It is supposed that this occurs because both DLE and SCC are triggered by ultraviolet radiation, which a) Causes DNA damage and genetic mutations, leading to skin cancer, and b) Produces apoptosis of keratinocytes, which are normally rapidly eliminated to avoid exposure of their intracellular, auto-antigenic components to the immune system but, in genetically susceptible individuals, predispose to discoid lupus.^5^ The development of SCC in an HDLE lesion can be difficult to diagnose, as HDLE already manifests as florid pseudoepitheliomatous hyperplasia. When the histological differentiation between HDLE and SCC is not clear, CD123 staining has been proposed as a diagnostic aid. CD123+ cells are more numerous at the dermal-epidermal junction of HDLE biopsies than in neoplastic- or other inflammatory dermatoses.^9^

The first-line agents in the management of CL are photoprotection, corticosteroids and antimalarials.^5^ Treatment of HDLE is frustrating for both the physician and the patient. Intralesional steroids provide only partial results. As second line agents, control has sometimes been achieved with retinoids, thalidomide and biological agents.^5^

Ertekin et al.^10^ described a case of association between hepatitis C and HDLE; the skin lesion was completely resolved after treatment with direct-acting antivirals. However, as HDLE is a rare presentation of CL, and the report is based on a single patient, little is known about the reality of this association.

Finally, HDLE is a warty form of cutaneous lupus, that can mimic many other conditions and is difficult to control. The risk of malignant transformation associated with the disfiguring potential of HDLE indicates the need for monitoring.

## Financial support

None declared.

## Authors’ contributions

Carolina Viza Amorim: Writing of the manuscript; data collection; final approval of the final version of the manuscript.

Maria de Lourdes Bialon Santana: Data collection; final approval of the final version of the manuscript.

Maria Leticia Cintra: Writing of the manuscript; effective participation in the research guidance; final approval of the final version of the manuscript.

Fernanda Teixeira: Writing of the manuscript; manuscript critical review; final approval of the final version of the manuscript.

## Conflicts of interest

None declared.
